# A 5-year retrospective study of *Actinomyces odontolyticus* bacteremia in the state of Qatar, case series

**DOI:** 10.1016/j.amsu.2022.103583

**Published:** 2022-04-02

**Authors:** Maisa Ali, Almurtada Razok, Hisham Ziglam

**Affiliations:** a Department of Infectious Diseases, Hamad Medical Corporation, P.O 3050, Doha, Qatar; b Department of Internal Medicine, Hamad Medical Corporation, P.O 3050, Doha, Qatar

**Keywords:** Case series, Infectious diseases, Anaerobes, Actinomyces, Bacteremia

## Abstract

**Introduction and importance:**

Manifestations of infection by *A. odontolyticus* include thoracic, abdominal, pelvic, and central nervous system disease. In the four decades following its isolation, more than 20 cases of invasive infections were reported in multiple geographic locations including the United States and Europe. As such, *A. odontolyticus* is an emerging bacterium and related research is encouraged for further characterization of its prevalence and clinical significance. Our case series represents the first case series about *A. odontolyticus* bacteremia in the state of Qatar.

**Methods:**

We are reporting 15 cases with isolated *A. odontolyticus* positive blood cultures at Hamad Medical Corporation, State of Qatar from 1/Jan/2016 to 1/Nov/2020. Electronic health records (EHR) of patients who were identified to have positive blood cultures were accessed and the demographics and other clinically related data were collected and mentioned in this manuscript, after obtaining the appropriate approval from the Corporation Medical Research Council (MRC).

**Outcomes:**

We are reporting 15 cases with isolated *A. odontolyticus* positive blood cultures at Hamad Medical Corporation, State of Qatar from 1/Jan/2016 to 1/Nov/2020.

**Conclusion:**

12 of the 15 reported cases were considered significant and received a complete course of antimicrobial therapy. The patients presented with a wide variety of clinical pictures and were of variable age.

## Highlights

• A. *odontolyticus* is an emerging bacterium with increasingly reported cases.
• It is an anaerobic, filamentous gram-positive bacterium.
• It is associated with cervical, thoracic, abdominal, and pelvic infections.
• It can affect a wide spectrum of patient population.

## 1 Introduction

Actinomyces species are Gram positive anaerobic, non-sporulating, non-acid fast, non-motile, irregularly staining bacterium. It colonizes oropharynx, urogenital and gastrointestinal tracts. Actinomyces is associated with a wide range of infections including dental caries, abscesses, intraabdominal and bloodstream infections. Recognized risk factors for the development of actinomycosis are Immunosuppression and local tissue damage. Actinomycosis is more common in men, except for pelvic disease in women as it is associated with intrauterine devices (IUDs) [1]. More than 30 species have been described, *Actinomyces israelii* being the most prevalent species [2]. *It* was the major human pathogen for actinomycosis identified in 1878 and fully delineated by Israel [3]. Since then, other species have been identified: including A. *naeslundii, A. viscosus, A. pyogenes, A denticolens, A. howellii, A. hordeovulneris,* and *A. meyeri*.

*Actinomyces odontolyticus (A. odontolyticus)* was isolated in 1958, from persons with advanced dental caries [3,4]. Datta S et al., reported 21 cases of actinomyces bacteraemia of which *A. odontolyticus* was identified in 5 of them, two cases of *A. meyeri* and one case of *A. israelli* [5]. Cone LA et al., described 25 patients with invasive infection caused by *A. odontolyticus*; Thirteen of them had pulmonary, cardiopulmonary or mediastinal disease, three had bacteraemia two of which were immunocompromised patients and one was a previously healthy patient [6].

Traditionally, isolation of actinomyces from any sterile specimen is always considered significant. However, in 21 reported cases of blood stream infections, only six cases received appropriate prolonged treatment [5]. Actinomyces species are susceptible to several antimicrobials, including penicillin, macrolide, and tetracyclines. Treatment of actinomycosis involves the combination of surgical drainage and prolonged courses of antibiotic therapy which most likely penicillin as a treatment of choice [7].

We are reporting 15 cases with isolated *A. odontolyticus* positive blood cultures at Hamad Medical Corporation, State of Qatar from 1/Jan/2016 to 1/Nov/2020. We aim to describe the characteristics, clinical significance, risk factors and treatment outcome. Up to date, there has been no case series or epidemiologic studies describing the clinical significance or characteristics of *A. odontolyticus* bacteremia in the state of Qatar*.* We hope that our manuscript will serve as a bridge to further research on this subject and will help in building both a national and international epidemiologic database for *Actinomyces odontolyticus.*

## 2 Methods

### 2.1 Registration

This research was registered in and approved by Hamad Medical Corporation Medical Research Council (MRC) with unique number **MRC-01-20-1119** and in accordance with the Declaration of Helsinki.

### 2.2 Study design

This is a multi-center, retrospective case series and the cases are non-consecutive.

### 2.3 Settings and timeframes

The patients were managed in in-patient wards inside three hospitals affiliated with the Hamad Medical Corporation in Qatar.

Data collection timeframe include patients with positive *A. odontolyticus* blood cultures from 1/Jan/2016 to 1/Nov/2020. Follow up period included six months post-discharge from the inpatient wards.

### 2.4 Follow up

The patients were followed for six months after discharge from the in-patient wards. Follow up methods included both telephone consultation and outpatient clinic visits.

This case series has been reported in line with the PROCESS Guideline.

## 3 Results

### 3.1 Participants

We present the data of 15 patients from our facilities who were found to have *A. odontolyticus* bacteremia between 1/Jan/2016 and 1/Nov/2020. Nine of the patients were females (60%). Median age of diagnosis was 45 years. 11 patients (73%) had comorbid conditions, five out of which had both hypertension and diabetes mellitus. 12 patients had fever (80%). Three patients (20%) had orofacial abnormalities (two in the form of dental caries and one with submental abscess). 12 patients (80%) received full courses of antibiotics, positive cultures for the remaining two were considered as contamination and therefore they did not receive a full course. The infectious disease team was consulted for six patients. Most of the patients received B-lactam antibiotics (60%). Maximum duration of antibiotics was 60 days and minimum duration was three days. One patient underwent surgical treatment in the form of incision and drainage for submental and mediastinal collections.

### 3.2 Outcomes and follow up

There was no deviation from the initial management plans.

One patient passed away while the other 14 recovered uneventfully with a case fatality rate of 6.6%.

### 3.3 Adherence and compliance

The patient adhered to the prescribed antibiotic regimens during their in-patient stay and after discharge. This was confirmed directly with the patients, and their surrogates where applicable.

### 3.4 Complications and adverse events

No complications were reported during the follow-up period and the patients tolerated the prescribed antibiotics with no remarkable adverse events.

## 4 Detailed presentation of cases

### 4.1 Case 1

A 37-year-old lady, five months pregnant, with background of sickle cell disease presented to the hospital in September 2016 with fever and right sided facial pain for three days’ duration. Her home medications were folic acid and as needed paracetamol. Aside from a temperature of 38.9 °C, vital signs were normal. Physical examination revealed right sided maxillary tenderness. Examination of the oral cavity, chest, heart, and abdomen was normal. No dental abnormalities were appreciated. White blood cell count was significantly elevated at 34.8 × 103/μL (Reference range 5–13 × 103/μL) and C-reactive protein was 81 mg/L (Reference range 0–5 mg/L). Chest X-ray was normal. Two out of four bottles of blood cultures were positive for *A. odontolyticus* which was sensitive to amoxicillin/clavulanate. The patient received two days of intravenous ceftriaxone and was discharged on oral amoxicillin/clavulanate for a total course of two weeks of antibiotics and her recovery was uneventful.

### 4.2 Case 2

A 1-day-old female neonate delivered uneventfully in March 2017 through low-segment Cesarean section, with no apparent congenital anomalies or dysmorphic features was found to have foul smelling meconium-stained amniotic fluid post-delivery. Her mother did not have any medical issues. Physical examination was normal, and her Apgar score was nine and ten at five and 10 min respectively. White blood cell count and C-reactive protein were normal. One bottle of pediatric blood culture was positive for *A. odontolyticus* which was sensitive to amoxicillin/clavulanate. She was started on intravenous amikacin awaiting the results of repeated blood cultures which were negative for any growth. The antibiotic was discontinued after three days, and she was discharged after completing her post-natal care uneventfully. Further follow up at two weeks post-discharge was unremarkable.

### 4.3 Case 3

A 76-year-old lady with background of heavy smoking, hypertension and chronic obstructive pulmonary disease presented in April 2017 with fever and cough productive of brownish sputum for three days. Her home medications included amlodipine, simvastatin, and fluticasone/salmeterol inhaler. Her temperature was 38.2 °C and her other vital signs were normal. Physical examination and X-ray of the chest revealed an area of consolidation in the right middle lobe. Oropharyngeal examination revealed poor oral hygiene and multiple dental caries; however, there was no evidence of a dental abscess. White blood cell count was 12 × 10^3^/μL. One bottle of blood culture was positive for *A. odontolyticus* which was sensitive to penicillin. She received three days of intravenous antibiotics which included ceftriaxone and azithromycin. She was prescribed oral amoxicillin/clavulanate for four more days and was discharged home. Her recovery was uneventful.

### 4.4 Case 4

A 58-year-old lady with background of spinocerebellar ataxia presented in August 2017 with fever and vomiting. Her home medications were pregabalin and mirtazapine. Her temperature was 38.2 °C and other vital signs were normal. Physical examination revealed tenderness in the right flank. White blood cell count was 13.2 × 10^3^/UL. Ultrasound of the abdomen showed a trace of right perinephric fluid. Blood culture was positive for *A. odontolyticus*. Urine culture was positive for *Escherichia coli* and *Klebsiella pneumoniae*. The patient received eight days of intravenous ceftriaxone and recovered without any major sequelae.

### 4.5 Case 5

A 73-year-old lady with background of hypertension, diabetes mellitus and Alzheimer's dementia presented in November 2017 with fever, chills, and fatigue for two days. Her home medications included metformin/sitagliptin, enalapril, and rosuvastatin. Vital signs were normal except for a temperature of 37.9 °C and a heart rate of 107 beats per minute. Physical examination of the chest and abdomen was normal. A detailed oropharyngeal examination was not documented. White blood cell count was 4 × 10^3^/UL and C-reactive protein 16 mg/L. Two bottles of blood cultures were positive for *A. odontolyticus*. Chest X-ray was normal. She received intravenous ceftriaxone for two days and was discharged on oral amoxicillin/clavulanate for seven days. Her recovery was uneventful.

### 4.6 Case 6

An 81-year-old lady with background of hypertension, diabetes mellitus and asthma presented in January 2018 with abdominal pain and vomiting for three days. Her home medications were metformin, amlodipine and salbutamol inhaler as needed. Vital signs were normal. Physical examination was remarkable for tenderness in the epigastric area. White blood cell count was 11.4 × 10^3^/UL and C-reactive protein was 149 mg/L. One bottle of blood culture was positive for *A. odontolyticus*. Ultrasound of the abdomen revealed acute acalculous cholecystitis. The patient received seven days of intravenous ciprofloxacin and metronidazole. Her recovery was uneventful.

### 4.7 Case 7

A 64-year-old gentleman with background of aortic stenosis, hypertension and diabetes mellitus complicated by diabetic foot status post recent surgical debridement and end-stage renal disease on regular hemodialysis presented in March 2018 with fever and lethargy which occurred during his dialysis session. His home medications were amlodipine, gliclazide and bisoprolol. His initial vital signs were remarkable for blood pressure of 80/64 mmHg and temperature of 38.4 C. Physical examination of the right foot revealed a deep ulcer in plantar aspect 4 × 3 cm in size covered by sloughed tissue. Other systems were normal. White blood cell count was 13. 9 × 10^3^/UL and C-reactive protein was 186 mg/L. Two bottles of blood culture were positive for *A. odontolyticus.* He received an intravenous renal-adjusted course of ciprofloxacin and clindamycin for ten days. He had an uneventful recovery and was discharged from the hospital after completion of antibiotics.

### 4.8 Case 8

A 1-month-old male infant with no chronic medical issues presented in April 2018 with fever and dry cough. He was delivered vaginally with no pre- or post-delivery complications. He was not receiving any medications at home. Vital signs were unremarkable except for a temperature of 38.1 °C. Physical examination of the oropharynx, chest and abdomen was normal. White blood cell count was 7.7 × 10^3^/μL. One bottle of pediatric blood culture grew *A. odontolyticus*. He received five days of intravenous ceftriaxone and recovered uneventfully.

### 4.9 Case 9

An 11-year-old male with background of chronic sinusitis presented in February 2018 with fever and headache. His temperature was 38.5 °C and other vital signs were normal. Physical examination was positive for erythema and congestion in the throat. Examination of other systems including the urogenital was normal. White blood cell count was 10.8 × 10^3^/μL. One bottle of pediatric blood culture was positive for *A. odontolyticus*. He received seven days of oral amoxicillin/clavulanate and recovered uneventfully.

### 4.10 Case 10

A 74-year-old lady with background of hypertension, diabetes mellitus, heart failure and chronic kidney disease presented in June 2018 with reduced level of consciousness and was found to have severe hyponatremia with serum sodium of 109 mmol/L (Reference range 136–145 mmol/L). Home medications included gliclazide, perindopril, metoprolol, and atorvastatin. Vital signs including the temperature were normal. Physical examination revealed a Glasgow coma scale of 13/15. Other systems were normal. White blood cell count was 7.6 × 10^3^/μL and C-reactive protein was 31 mg/L. One bottle of blood culture was positive for *A. odontolyticus.* As the patient did not have clinical signs of infection, she was not started on antibiotics. Repeated blood cultures were negative. The patient was discharged home after improvement in serum sodium.

### 4.11 Case 11

A 32-year-old gentleman with no previous medical history, presented in September 2018 with fever and right-sided facial swelling. The patient had undergone removal of wisdom tooth #48 seven days prior. Vital signs were remarkable for a temperature of 38.2 °C. Physical examination revealed swelling and fluctuance in the right submental area. White blood cell count was 22 × 10^3^/μL and C-reactive protein was more than 500 mg/L. Computed tomography scan of the neck with contrast revealed a submental abscess with extension to the mediastinum and abscess formation with signs of mediastinitis. The largest mediastinal pocket was sized as 7 × 4 cm. The patient underwent incision and drainage of the submental abscess and mediastinoscopy with drainage of superior and anterior mediastinal abscesses. Blood cultures were positive for *A. odontolyticus*. Pus cultures from the submental and mediastinal abscesses were positive for *A. odontolyticus*. The isolates were sensitive to amoxicillin/clavulanate. He initially received piperacillin/tazobactam for three days which was later changed to intravenous clindamycin. He received antibiotics for a total duration of 28 days. He recovered successfully with no major sequelae.

### 4.12 Case 12

A 56-year-old gentleman with metastatic pancreatic carcinoma admitted in December 2018 with persistent abdominal pain. Last dose of chemotherapy was two weeks prior to his presentation. He had received gemcitabine and paclitaxel. He underwent celiac plexus block for his abdominal pain. Two days later, the patient developed signs of sepsis with fever of 38.9 °C and hypotension. Physical examination revealed a cachectic gentleman with scleral and cutaneous icterus. His labs revealed deterioration in serum bilirubin and alkaline phosphatase. White blood cell count was 14.9 × 10^3^/μL and C-reactive protein was 249 mg/L. Blood cultures were positive for *A. odontolyticus* which was sensitive to amoxicillin/clavulanate. The patient was diagnosed as ascending cholangitis and was initially started on piperacillin/tazobactam which was later escalated to meropenem and vancomycin due to clinical and laboratory deterioration. Ten days later, the patient's status deteriorated further, and he developed cardiac arrest. Unfortunately, the patient passed away. He received ten days total duration of antibiotics.

### 4.13 Case 13

A 2-year-old female baby with background of D-2-hydroxyglutaric aciduria and seizure disorder presented in February 2019 with vomiting. Her home medications included levetiracetam, phenobarbitone and prednisolone. Vital signs were remarkable for a temperature of 38.2 °C. Physical examination was otherwise normal. White blood cell count was 8.9 × 10^3^/μL. One bottle of pediatric blood culture was positive for *A. odontolyticus*. She received four days of intravenous ceftriaxone which was stopped after fever had resolved. Her vomiting stopped and she was discharged home. The patient remained stable and asymptomatic at two weeks follow up.

### 4.14 Case 14

A 2-year-old male baby with no chronic medical issues, presented in May 2019 with fever and truncal rash. His temperature was 38.9 °C. Other vital signs were normal. Physical examination revealed erythema and congestion in the throat and pink-colored confluent patches on the torso. Other systems were normal. White blood cell count was 6.4 × 10^3^/μL and C-reactive protein was 32 mg/L. One out of four bottles of pediatric blood culture was positive for *A. odontolyticus*. She was diagnosed as roseola infantum and was not started on antibiotics. Fever and rash subsided with ibuprofen and the patient was discharged home uneventfully. The patient remained stable and asymptomatic at three weeks follow up.

### 4.15 Case 15

A 45-year-old lady with background of hypertension, diabetes mellitus and chronic kidney disease admitted in December 2019 due to urinary tract infection and worsening in her kidney function. Three days after finishing her seven-day course of intravenous ertapenem, the patient developed fever of 38.4 °C. Other vital signs were normal. Physical examination revealed tenderness in the hypogastric region. Oropharyngeal examination by a dentist revealed several decayed dental roots. White blood cell count was 7.6 × 10^3^/μL and C-reactive protein was 80 mg/L. Blood culture was positive for *A. odontolyticus* and urine culture was positive for extended-spectrum beta-lactamase producing *Escherichia coli*. The patient received two weeks of intravenous ertapenem, followed by two weeks of intravenous ceftriaxone which was subsequently switched to oral amoxicillin/clavulanate to complete a total duration of antibiotics of 60 days. She recovered successfully.

## 5 Discussion

*A. odontolyticus* normally a commensal organism found in the mouth, was first isolated from dental caries in 1958 [8]. A variety of *A. odontolyticus* culture-positive infections have been documented in the literature. However, less commonly recognized as a pathogen than *A. israelli.* Most cases with invasive disease have presented with pulmonary, cardiopulmonary or mediastinal pathology [[8], [9], [10], [11], [12], [13], [14], [15]]. The incidence of *Actinomyces odontolyticus* bacteremia is less common. As with all other actinomycotic diseases, *A. odontolyticus* infections are endogenous, originating from mucous membranes and tend to grow predominantly on the surface of the tongue in supra and subgingival regions [4], which may account for a normal dental finding of the patient. The capacity of actinomycetes to colonize mucosal surfaces and dentine appears to depend on two distinct fimbriae, type 1 and type 2, that bind preferentially to salivary acidic proline-rich proteins and to Statherian, or to β-linked galactose or galactosamine structures on epithelial or bacterial surfaces, respectively [16]. *Actinomyces* bacteremia, once rare is now more commonly reported due to modern culture techniques. Not all positive cultures are clinically significant and relevant, as they can present as transient bacteremia or contamination as encountered in some of our patients (Cases 2, 10 and 14). We report 15 cases with positive *A. odontolyticus* blood cultures at Hamad Medical Corporation, State of Qatar. Our patients with bacteremia fall into one of two groups. The first group consists of pediatric patients with unremarkable co-morbidities apart from two children one of whom had chronic sinusitis and the other suffered from a neurometabolic disorder. The second group includes older adults, often with co-morbidities that pre-dispose to infection, such as diabetes mellitus or hypertension (Table 1). Fever was the main presenting sign and symptom in 12 patients (80%). Our reported cohort is different from patients reviewed by Cone et al. who found that infections caused by *A. odontolyticus* more frequently affecting immunosuppressed patients, predominantly middle-aged males [6]. Although it is an oral commensal organism, *A. odontolyticus* causes infection rarely and rarely shows resistance to antibiotics (Table 2). The organism is more likely to cause infection in several specific sub-groups than in others. Our cohort highlights the pathology that can be induced by *A. odontolyticus* even in the absence of immunosuppression [17].

**Table 1:** Reported cases of Actinomyces odontolyticus bacteraemia in Hamad Medical Corporation between 1/Jan/2016 to 1/Nov/2020.

Gender	Age	Year of diagnosis	Clinical presentation	Fever	Antibiotics	Type of antibiotic	Duration of antibiotics	ID team consultation	Co-morbidities
Female	37 years	2016	Fever and facial pain	Yes	Yes	Amoxicillin/Clavulanate	14 days	Yes	SCD
Female	1 day	2017	Meconium stained amniotic fluid	No	Yes	Amikacin	3 days	No	None
Female	79 years	2017	Fever and cough	Yes	Yes	Amoxicillin/Clavulanate	7 days	No	COPD
Female	58 years	2017	Fever and vomiting	Yes	Yes	Ceftriaxone	8 days	No	Spinocerebellar ataxia
Female	73 years	2017	Fever and chills	Yes	Yes	Amoxicillin/Clavulanate	9 days	No	HTN, DM
Female	81 years	2018	Vomiting	No	Yes	Ciprofloxacin + Metronidazole	7 days	Yes	HTN, DM
Male	64 years	2018	Fever and lethargy	Yes	Yes	Ciprofloxacin + Clindamycin	10 days	Yes	HTN, DM, ESRD
Male	1 month	2018	Fever and cough	Yes	Yes	Ceftriaxone	5 days	No	None
Male	11 years	2018	Fever and headache	Yes	Yes	Amoxicillin/Clavulanate	7 days	No	Chronic sinusitis
Female	74 years	2018	Reduced level of consciousness	No	No	None		No	HTN, DM, HF, CKD
Male	32 years	2018	Fever and facial swelling	Yes	Yes	Clindamycin	28 days	Yes	None
Male	56 years	2018	Sepsis	Yes	Yes	Meropenem + Vancomycin	10 days	Yes	Pancreatic cancer
Female	2 years	2019	Vomiting	Yes	Yes	Ceftriaxone	4 days	No	D-2-hydroxyglutaric aciduria
Male	2 years	2019	Fever	Yes	No	None		No	None
Female	45 years	2019	Fever	Yes	Yes	Amoxicillin/Clavulanate	60 days	Yes	HTN, DM, CKD

**Table 2:** Minimal inhibitory concentrations (MICs) of selected antibiotics against A. odontolyticus, including interpretations and breakpoints, as reported by the AMRHAI reference unit, PHE Colindale

Antibiotics	MIC	S/I/R	Breakpoint
Co-amoxiclav	0.125	S	8 and 16
Cefotaxime	0.25	S	8 and 16
Ceftriaxone	0.25	S	8 and 32
Imipenem	0.064	S	2 and 4
Co-trimoxazole	0.125	S	2
Clarithromycin	≤0.016	S	2 and 4
Linezolid	0.25	S	4
Ciprofloxacin	4	R	1 and 2
Moxifloxacin	2	I	1 and 2
Doxycycline	0.064	S	1 and 4
Minocycline	≤0.016	S	1 and 4

In conclusion, clinicians of all specialties need to be aware of the rising number of reports of *Actinomyces* species bacteremia due to widespread availability of molecular identification techniques, including MALTI-TOF. Further research to look more closely at *Actinomyces* spp. isolated from other culture types will help to elucidate the true significance of these isolates. Furthermore, more studies are needed to determine guidelines for treating these resilient microbes.

### 5.1 Strengths of the study

This is the first case series about *A. odontolyticus* bacteremia in the state of Qatar, with full description of the patients’ demographics and presentation.

### 5.2 Limitations of the study

Relatively small number of available patients.

This case series has been reported in line with the PROCESS Guideline [18].

## Availability of data and materials

The datasets used and/or analyzed during the current study are available from the corresponding author on reasonable request.

## Provenance and peer review

Not commissioned, externally peer-reviewed.

## Sources of funding

This article did not receive any specific grant from funding agencies in the public, commercial, or not-for-profit sectors.

Open access funding will be provided by Qatar National Library (QNL).

## Ethical approval

Approval from the Hamad Medical Corporation Medical Research Council was obtained prior to submission of this manuscript. Manuscript reference MRC-01-20-1119**.**

## Consent

Written informed consents were obtained from the patients for publication of this case series and the accompanying information. Copies of the written consents are available for review by the Editor-in-Chief of this journal on request.

## Author contribution

MA and AR performed literature review and wrote the original draft of the manuscript. HZ supervised the writing process and revised the manuscript. All authors approved the final version for submission.

## Registration of research studies

This research was registered in and approved by Hamad Medical Corporation Medical Research Council (MRC) with unique number **MRC-01-20-1119** and in accordance with the Declaration of Helsinki.

## Guarantor

Dr. Almurtada Razok.

## Declaration of competing interest

None to be declared.
